# Evaluation of Bioceramic Putty in Pulpotomy of Immature Permanent Molars With Symptoms of Irreversible Pulpitis

**DOI:** 10.7759/cureus.31806

**Published:** 2022-11-22

**Authors:** Ibrahim Alnassar, Mohamed K Altinawi, Mohamad Salem Rekab, Hasan Alzoubi, Imad Katbeh

**Affiliations:** 1 Department of Pediatric Dentistry, Damascus University, Damascus, SYR; 2 Department of Endodontic, Damascus University, Damascus, SYR; 3 Department of Pediatric Dentistry and Orthodontics, Peoples' Friendship University of Russia (RUDN University), Moscow, RUS

**Keywords:** bioceramic putty (well-root pt), mta, irreversible pulpitis, pulpotomy, immature permanent molars

## Abstract

Purpose

This study aimed to evaluate the effectiveness of both mineral trioxide aggregate (MTA) and bioceramic putty (Well-Root PT) in the pulpotomy of immature permanent molars diagnosed with symptoms of irreversible pulpitis.

Materials and methods

The study included 30 immature permanent molars with symptoms of irreversible pulpitis in 30 healthy children aged six to eight years. They were randomly distributed into the following two groups according to the material used: group 1 included 15 first permanent molars capped by MTA and group 2 included 15 first permanent molars capped by bioceramic putty. Clinical and radiographical evaluations of the treatment results were made after one week, three months, six months, nine months, and 12 months.

Results

The success rate in the bioceramic putty group was 93.3% clinically and radiographically after a 12 months follow-up, whereas in the group that underwent MTA treatment no cases of failure were registered with a 100% success rate. No statistical differences were observed between groups (p=0.309). The dentin bridge was formed in 60% of the MTA group and 33.3% of the bioceramic group without any statistically significant differences (p=0.272) after a 12 months follow-up.

Conclusion

Pulpotomy using biocompatible materials (MTA and bioceramic putty) on immature permanent molars with symptoms of irreversible pulpitis is considered acceptable and effective.

## Introduction

Diagnosis and treatment of carious and posttraumatic immature permanent molars are considered a challenging aspect of daily clinical routine [1], where constant irritation of the pulp without treatment leads to irreversible pulpitis and necrosis followed by apical periodontitis, which hinders the natural root development and complete formation thus reducing the prognosis of retaining these teeth in the oral cavity [2,3]. Therefore when treating immature permanent molars, the main goal is to preserve the vitality of the pulp to guarantee natural root development [4].

For many years vital pulp therapy focused on immature teeth that don't show signs of irreversible pulpitis to guarantee natural root development, but the concept of vital pulp therapy expanded to include immature permanent molars affected by irreversible pulpitis [5]. The response of dental pulp differs between children and adults. Where the pulp of immature teeth is characterized by natural defenses and various vascularity thus increasing the pulp's resistance to bacterial invasion for a long period leading to an increased success rate for vital pulp therapy in immature teeth compared to mature teeth [6].

Recently, pulpotomy with biocompatible materials has been used as a conservative and simple technique in the management of immature permanent molars affected by irreversible pulpitis relying on the ability of the remaining radicular pulp to recover after the removal of the inflamed coronal tissues where this technique showed a high and acceptable success rate [7-9] as studies noted a weak relationship between pulp's histological status and the patient's symptoms [10]. In multiple studies, MTA showed a high success rate in the treatment of immature permanent molars affected by irreversible pulpitis [11,12].

In 2007 a group of Canadian researchers introduced bioceramic, a calcium silicate-based material that is ready to use with no need for mixing. This material is available in the following three forms: bioceramic root repair material putty (BC RRM, fast set putty), BC RRM past (a syringable past), and BC sealer. Bioceramic putty showed high success rates in the pulpotomy of immature permanent molars affected by irreversible pulpitis or traumatic injuries [13,14].

Both MTA and bioceramics have similar compositions, but bioceramic material contains titanium oxide and calcium phosphate, in addition to the absence of aluminum in its composition. Bioceramics can release a high percentage of calcium ions early while maintaining this high percentage for 28 days, in contrast to the mineral trioxide, which showed a lower ability to release calcium ions more slowly [15]. Therefore, this study was conducted to compare the effectiveness of both MTA and bioceramic putty (Well-Root PT) in the pulpotomy of immature permanent molars with symptoms of irreversible pulpitis.

## Materials and methods

A randomized controlled trial was conducted in 30 immature permanent molars to study the pulpotomy effectiveness in case of irreversible pulpitis. The study protocol was approved by the Scientific Research and Postgraduate Board and Ethics Committee of Damascus University, Damascus, Syria. All procedures followed the ethical standards of the institution and the 1964 Helsinki Declaration and its later amendments or comparable ethical standards. This research was registered in the Australian New Zealand Clinical Trials Registry under the number: ACTRN12621001631897. A detailed information sheet in simple nontechnical language was provided in advance, and parents/guardians were requested to sign an informed consent.

**Figure 1:**
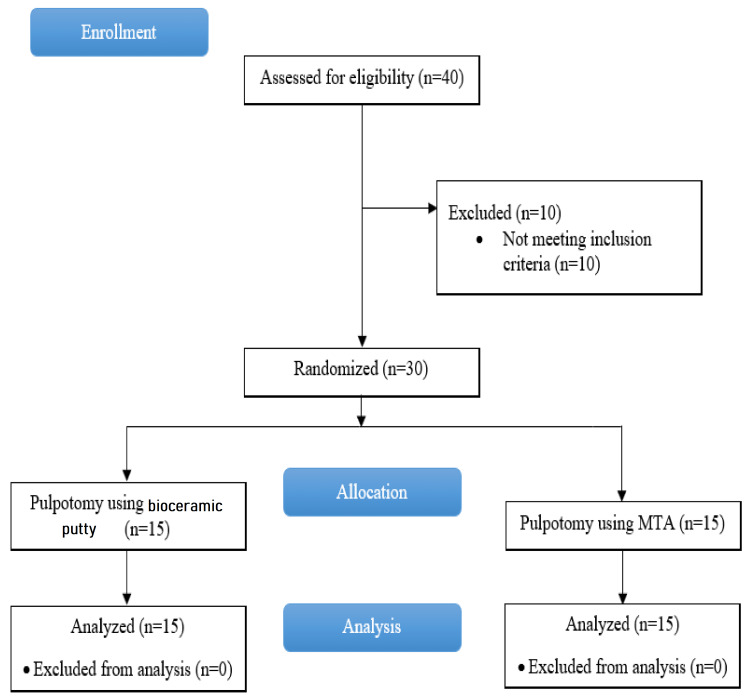
Consort flow diagram. MTA: mineral trioxide aggregate

The sample size was calculated using G*Power software (PS Power and Sample Size Calculation program, version 3.0.43, https://biostat.app.vumc.org/wiki/Main/PowerSampleSize) based on a previous study [16]. Sample size calculation produced a required sample size of 15 immature permanent molars per group to detect a significant difference (90% power, two-sided 5% significance level). The studied sample was randomly distributed (at http://www.randomization.com//) into the following two groups: group 1 represented the control group which was capped by MTA, and group 2 represented the experimental group which was capped by bioceramic putty (Figure 1). A double-blind was also adopted in this study so that both the patient and the examiner would not know about the applied material.

Inclusion criteria

Children of ages ranging between six and eight years, presence of an extended carious lesion with symptoms of irreversible pulpitis, absence of signs or symptoms of pulpal necrosis (fistula, tooth mobility, swelling), absence of radiographic signs (internal or external resorption, periapical lesion or bifurcation lesion), cooperative children, and restorable teeth were included in the study.

Exclusion criteria

Children with special needs care, teeth with crowns that can't be isolated, children with systemic diseases (immune, cardiovascular, or those that underwent cardiovascular surgery), children with bad oral hygiene, and uncontrolled pulp hemorrhage were excluded from the study.

Work procedure

All dental treatments were provided in the Department of Pediatric Dentistry, Faculty of Dentistry, Damascus University. After applying the local anesthetic gel, lidocaine was administered 2% with 1:80000 epinephrine, and isolation was done with a rubber dam. Enamel and dentin were removed by using a high-speed diamond bur with a water-cooled handpiece, taking into account the removal of the caries area near the pulp by using an excavator from the periphery towards the center to reduce the amount of bacterial contamination. The pulp chamber access was refined with Endo-Z (Ballaigues, Switzerland: Dentsply Maillefer) with water cooling and then the remainder of the coronal pulp was removed by a sharp excavator. Hemostasis was achieved with cotton pellets moistened with 2.5% sodium hypochlorite for two minutes, and the process is repeated, if necessary, until the bleeding stops within 10 minutes [17]. If hemostasis was not achieved, the case was excluded and pulpectomy treatment was done. Then apply a BC putty (Well-Root PT) or MTA angelus depending on the group selected (the studied sample was randomly distributed at http://www.randomization.com, a double-blind was also adopted in this study so that both the patient and the examiner would not know about the applied material). A base layer of glass ionomer cement was applied (Fuji IX®; Tokyo, Japan: GC Corporation) and then the tooth was restored with Tetric N-Ceram resin composite (Schaan, Liechtenstein: Ivoclar Vivadent) in class I cavities or a stainless-steel crown (Seoul, Korea: KidsCrown) in class II cavities.

Clinical and radiographic assessment

**Figure 2:**
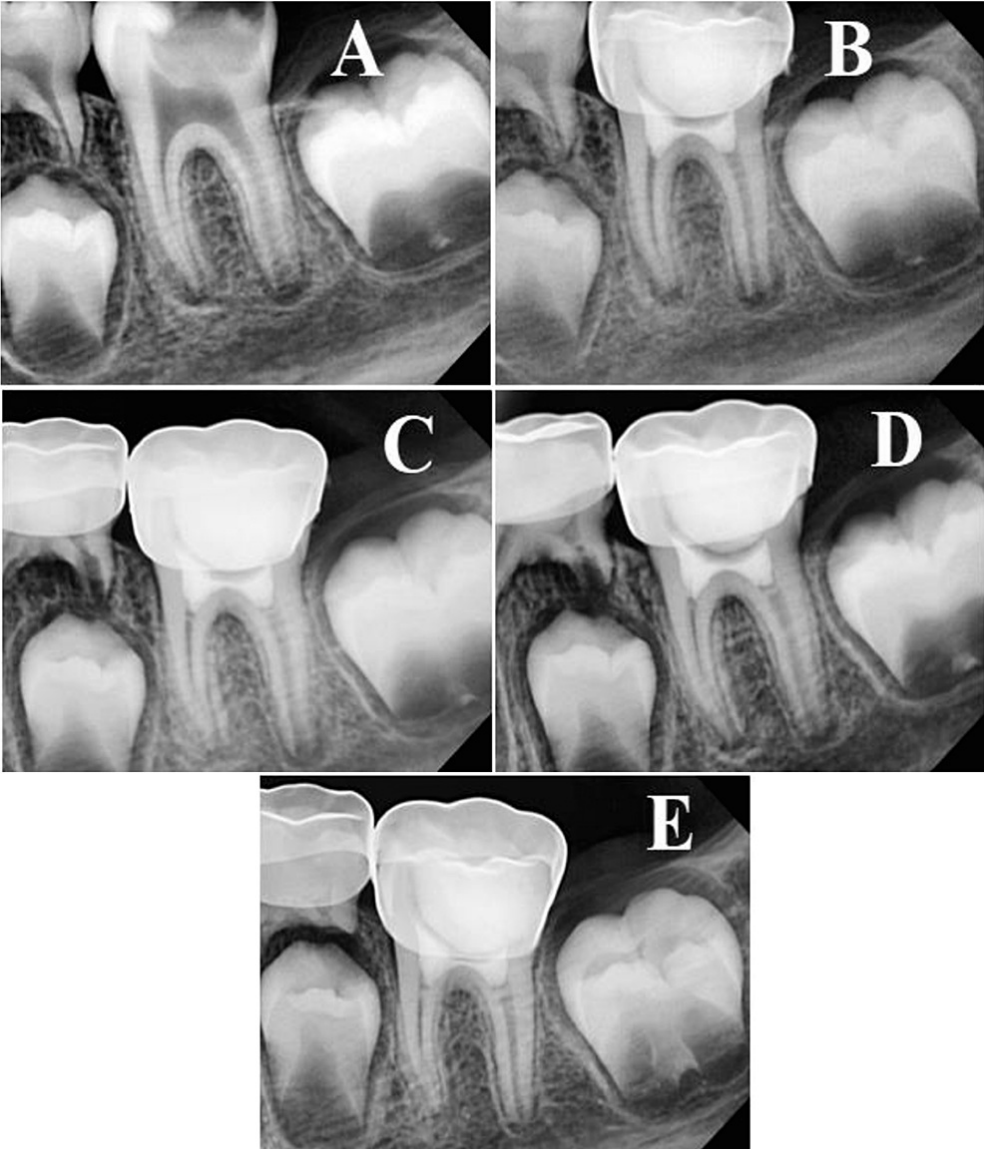
MTA follow-up (A) diagnostic image, (B) one-week follow-up, (C) three months follow-up, (D) six months follow-up, and (E) 12 months follow-up. MTA: mineral trioxide aggregate

**Figure 3:**
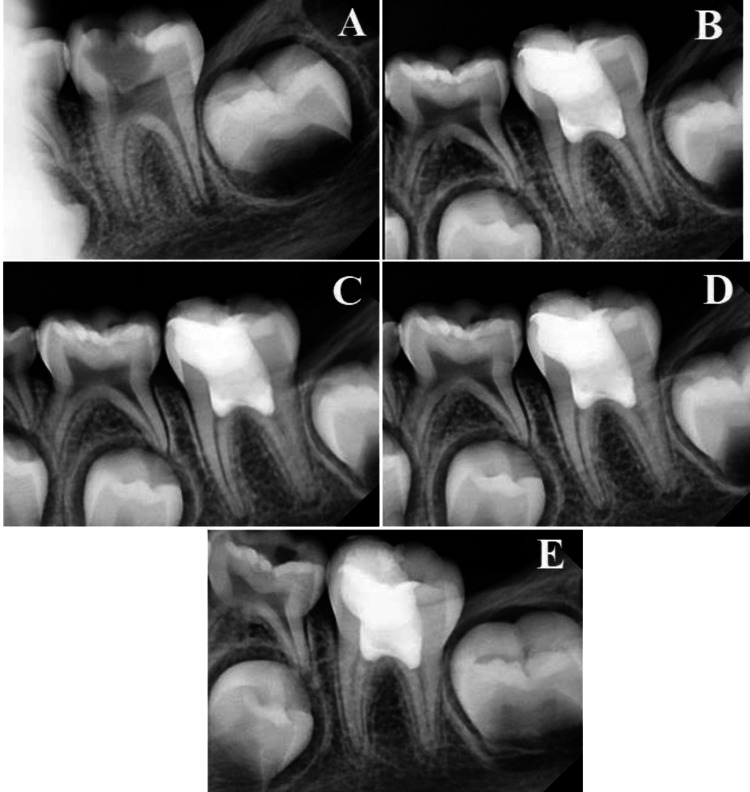
Bioceramic putty follow-up (A) diagnostic image, (B) one-week follow-up, (C) three months follow-up, (D) six months follow-up, and (E) 12 months follow-up.

The evaluation was made after one week, three, six, nine, and 12 months by two specialists with no prior knowledge of the materials used. Treatment was considered successful with the absence of spontaneous pain, pain on percussion, and the absence of swelling and fistula. Treatment was considered radiographically successful with the absence of widening of the periodontal ligament, and internal or external root resorption in addition to root development and maturation. The assessment also included hard tissue bridge formation and canal obliteration (Figures 2A-2E and Figures 3A-3E).

Statistical analyses

Chi-square test was used to assess the success and failure rates between the groups. The IBM SPSS Statistics software for Windows, version 20.0 (Armonk, NY: IBM Corp.) was used for data analysis. The results were considered to be statistically significant at p≤0.05.

## Results

The study sample included 30 immature lower permanent molars divided equally into two groups. The ages of the children ranged from six to eight years. The average age in the first group was 7.39±0.57 whereas in the second group it was 7.29±0.53 (Table 1).

**Table 1:** Basic sample characters. MTA: mineral trioxide aggregate

Group	Sex	N (%)	Age (mean±SD)
MTA	Male	9 (60%)	7.39±0.57
	Female	6 (40%)	
Bioceramic	Male	6 (40%)	7.29±0.53
	Female	9 (60%)	

After 12 months of observation, success rate reached up to 100% clinically and radiographically in the MTA group. On the other hand, the success rate was 93.3% in the bioceramic group. No statistical differences were observed between groups (p=309). The hemostasis time was similar in the MTA and bioceramic groups (7.43±1.49 minutes and 7.36±1.39 minutes, respectively). The dentin bridge was formed in 60% of subjects in the MTA group and 33.3% of subjects in the bioceramic group without any statistically significant differences between the groups (Tables 2, 3).

**Table 2:** Comparisons of success and failure rate during observation periods. MTA: mineral trioxide aggregate

Observation periods		Group	Success	Failure	p-Value
1 week	Clinical	MTA	100%	0%	-
		Bioceramic	100%	0%	
	Radiographic	MTA	100%	0%	-
		Bioceramic	100%	0%	
3 months	Clinical	MTA	100%	0%	0.309
		Bioceramic	93.3%	6.7%	
	Radiographic	MTA	100%	0%	0.309
		Bioceramic	93.3%	6.7%	
6 months	Clinical	MTA	100%	0%	0.309
		Bioceramic	93.3%	6.7%	
	Radiographic	MTA	100%	0%	0.309
		Bioceramic	93.3%	6.7%	
12 months	Clinical	MTA	100%	0%	0.309
		Bioceramic	93.3%	6.7%	
	Radiographic	MTA	100%	0%	0.309
		Bioceramic	93.3%	6.7%	

**Table 3:** Comparisons of dentin bridge formation during observation periods. MTA: mineral trioxide aggregate

Observation periods	Group	Presence	Absence	p-Value
3 months	MTA	60%	40%	0.272
	Bioceramic	33.3%	66.7%	
6 months	MTA	60%	40%	0.272
	Bioceramic	33.3%	66.7%	
12 months	MTA	60%	40%	0.272
	Bioceramic	33.3%	66.7%	

## Discussion

The results of pulpotomy are affected by several factors such as diagnosis, hemostatic agents, coronal seal, and the biocompatible material used [18]. Leaving carries with no treatment leads to its extension towards the pulp eventually causing irreversible pulpitis which indicates the complete removal of pulp tissues [19]. However, pulpectomy in immature permanent molars affected by irreversible pulpitis is considered a challenge due to their brittleness and thin walls of their roots which could cause root fractures in addition to the open apex, making it difficult to achieve a good apical seal [20].

Therefore, pulp vitality in immature molars should be preserved to guarantee root development, completion, and closure of the apex. In such cases, a vital pulpotomy is considered the best option due to its ability to preserve the vitality of the pulp, in addition to this being an easy procedure, relatively inexpensive, and fast procedure for shorter treatment sessions which is an important consideration in the management of pediatric patients. Also, when compared to root canal treatment, it requires fewer radiographs and is painless [19-21]. It also preserves the immunological functions of the pulp and the integrity of the remaining tooth structure [22].

The study sample consisted of 30 immature lower permanent molars randomly distributed into two groups, lower immature permanent molars were chosen for this study to avoid anatomical overlapping in the maxilla and to unify the parameters resulting from the type of molars. The sample consisted of molars diagnosed with irreversible pulpitis based on the patients' symptoms that included spontaneous and continuous pain that requires giving the child analgesics and on clinical carious exposure. Pulp vitality tests were not used due to their unreliability in immature molars [6].

A period of 5 to 10 minutes to reach hemostasis after removing the coronal tissues was agreed on to determine the eligibility of the case, if hemostasis couldn't be reached within less than 10 minutes the case was excluded and transferred to a pulpectomy, this increased bleeding indicates severe inflammation based on Wolter's classification [6].

A concentration of 2.5% of sodium hypochlorite was used as an irrigation solution after removing the coronal pulp, based on the recommendations of the American Association of Endodontists, which recommended the use of sodium hypochlorite with a concentration that ranges between 0.5% and 5.25% in the management of vital pulp tissues. Sodium hypochlorite is considered safe with no toxic effects on the cells of the pulp or their cytodifferentiation, also it didn't show any negative effect on hard tissue deposition [5].

The quality of the restoration also plays an important role in the outcome of the treatment [23]. Stainless steel crowns were used as final restorations due to their high ability to achieve a coronal seal and their longevity, in addition, treatment was completed in one visit and thus microleakage was minimal [24].

MTA is considered a standard material in vital pulp therapy due to its high biocompatibility and success rate [25], therefore, it was used in this study, and its effects were compared to those of bioceramic putty (Well-Root PT), which has high biocompatibility and success rates and is greatly capable of achieving a coronal seal in addition to its hard tissue deposition inducing ability [18].

After a one-year follow-up, the clinical and radiologic success rate of bioceramic putty (Well-Root PT) reached 93.4%, with one case of failure registered after three months (where the appearance of pain on the bite was observed, in addition to a large periapical translucency), which might be caused by incomplete removal of the infected pulp tissues during treatment. Whereas MTA had a 100% clinical and radiologic success rate after a one-year follow-up. Clinical success was determined by the absence of spontaneous pain and pain on percussion and the absence of a fistula, whereas radiologic success was determined by the complete root development and closure of the apex and absence of bifurcation and periapical lesion and absence of internal or external resorption. Hard tissue deposition and pulp canal obliteration were also radiographically assessed, seven molars from the MTA group witnessed dentinal bridge formation and two cases had pulp canal obliteration, whereas the group that underwent bioceramic putty (Well-Root PT) treatment witnessed five cases of dentinal bridge formation and one case of root canal obliteration. These cases were classified as successful.

The results of this study were close to those of Zhe and Jieying, who studied the effectiveness of iRoot BP Plus in the partial pulpotomy of mature permanent molars affected by irreversible pulpitis. They noted a success rate of 86.96% after three years of observation with a difference in the type of treatment used, where they applied partial pulpotomy on mature permanent teeth, whereas we treated immature permanent teeth with a standard pulpotomy [26].

The results of this study were also similar to those of Guan et al., who studied the effectiveness of bioceramic (iRoot BP Plus) in the treatment of permanent molars affected by irreversible pulpitis in patients of ages between 6 and 20 years, where they reached a success rate of 91.2% after one year of observation [13].

The results of this study are in accord with Qian et al., who compared bioceramic putty iRoot and MTA in the pulpotomy of permanent molars affected by irreversible pulpitis, the success rates of iRoot bioceramic putty reached 94.4% and MTA had a 100% success rate after one year of observation [27]. We are also in accord with Qudeimat et al., who had a 100% success rate with MTA after the observation that ranged from 18.9 to 73.6 months [12]. We are also in accord with Gomez et al., who had a 100% success rate with MTA in their study after a year of observation [28].

Limitations

This study was done on immature molars with symptoms of irreversible pulpitis, and this is the major limitation of the study. This is because of the inability to determine the extent of inflammation to the radicular pulp and to rely on the time of hemostasis and child symptoms.

## Conclusions

Both MTA and bioceramic putty (Well-Root PT) showed promising results in the pulpotomy of immature permanent molars with symptoms of irreversible pulpitis, making it an acceptable conservative option while taking into consideration, the necessity for long-term studies with a larger sample size to assess the risk, success, and the benefits of these materials. In addition to histological studies to further investigate the pulp's response in such cases.
